# Case report: narcolepsy type 2 due to temporal lobe glioma

**DOI:** 10.1097/MD.0000000000021002

**Published:** 2020-07-10

**Authors:** Yuangao Liao, Yan He, You Yang, Xiaojie Li, Fengzhen Huang

**Affiliations:** a Department of Neurology and Sleep Medical Center, the First People's Hospital of Chenzhou; b Department of Radiology, the First People's Hospital of Chenzhou; c Pathological Diagnosis Center, the First People's Hospital of Chenzhou, China.

**Keywords:** excessive daytime sleepiness, glioma, hippocampus, narcolepsy type 2

## Abstract

**Rationale:**

The orexin projection system includes the lateral hypothalamus, reticular activating structure, and ventrolateral preoptic nucleus, and this system is related to the pathogenesis of narcolepsy. Here, we report a case of narcolepsy type 2 caused by hippocampal glioma of the right temporal lobe.

**Patient concerns:**

A 44-year-old male farmer complained of excessive daytime sleepiness (EDS) over the past 3 months and more.

**Interventions:**

The lesion of the right anteromedial temporal lobe was removed and its pathological examination was carried out.

**Outcomes:**

General examination showed no abnormalities of his heart, lungs, or abdomen. Neurological examination showed no positive sign. The blood routine and biochemical examination were normal. He scored 7 on the Pittsburg sleep quality index, 16 on the Epworth sleepiness scale, 52 on the self-rating anxiety scale, and 48 on the self-rating depression scale. The multiple sleep latency test data showed 2 periods of sleep-onset rapid eyes movement period across 4 successive tests; the average sleep latency was under 8 minutes, and the rapid eyes movement latency was under 7 minutes. Lesion of glioma in hippocampus area of the right anteromedial temporal lobe was confirmed through magnetic resonance imaging, magnetic resonance spectroscopy, and histological examination. After surgical removal of the glioma from the hippocampus area of the right anteromedial temporal lobe, the patient's EDS symptoms disappeared immediately. He scored 3 on the Epworth sleepiness scale. During our follow-up three months later, he remained well with no complications.

**Diagnosis:**

We diagnosed the patient with narcolepsy type 2 according to the 3rd Edition of International Classification of Sleep Disorders (ICSD-3).

**Conclusion:**

The patient suffered from EDS and was diagnosed with narcolepsy type 2. The narcolepsy type 2 was linked to glioma of the hippocampus area. The hippocampus might be another part of regulating the sleep-arousal pathway, and the glioma secretion might interact with the orexin projection system.

## 1 Introduction

Narcolepsy is a rare central hypersomnia with an estimated prevalence of 0.02%, and it exists in 2 forms, narcolepsy type 1 and type 2.^[1]^ Narcolepsy type 2 is characterized by excessive daytime sleepiness (EDS) and pathological manifestation of rapid eyes movement sleep (REM sleep) (hypnagogic hallucinations, sleep paralysis, or sleep onset REM sleep).^[2]^ The orexin projection system includes the lateral hypothalamus, reticular activating structure, and ventrolateral preoptic nucleus, and it is related to the pathogenesis of narcolepsy.^[3]^ It has been reported that the volume of the hippocampus is related to the alertness and somnolence of the patients with low-ventilation sleep apnea-hypopnea syndrome^[4,5]^ and that the broadening of the hippocampus fissure is related to the severity of the sleep apnea-hypopnea syndrome.^[6]^ However, the pathogenesis of narcolepsy type 2 is still not clear. Here, we report a case of narcolepsy type 2 caused by hippocampal glioma of the right temporal lobe.

## 2 Report of case

A 44-year-old male farmer suffered from EDS over the previous 3 months and more. He was admitted to our hospital. He tended to be somnolent during the daytime and occasionally fell asleep when doing farm work, even though his sleep at night sleep was longer and better than before. He experienced slight snoring with no hypnagogic hallucination or sleep paralysis. He did not complain of cataplexy, hyperphagia, or hypersexuality. He had no headaches, dizziness, paralysis, numbness, or convulsions. He had no past medical history of mental stimulation, head trauma, drug abuse, hypertension, or diabetes. His family and relatives had no similar EDS complaints. He had received no treatment for his EDS symptoms. At admission, he was fully conscious but short of energy. General examination showed no abnormalities of his heart, lungs, or abdomen. Neurological examination showed no positive sign.

The blood routine and biochemical examination were normal. Serum thyroid-stimulating hormone (TSH) was slightly increased (0.229 μIU/ml), whereas free triiodothyronine (FT3) and free tetraiodothyronine (FT4) were normal. The serum was negative for antibodies against hepatitis C, syphilis, and AIDS. Electrocardiography showed sinus tachycardia and left axis deviation with no abnormalities in QRS intervals or QT intervals or ST-T changes. Chest computed tomography examination showed solitary nodules in the right middle lung, of which the size, location, and shape were the same as they had been 6 months earlier. Color Doppler ultrasound examination of the digestive system, urinary system, and carotid vertebral artery was all normal. No abnormalities in the form, structure, valve activities, or functionality were found in heart Doppler ultrasound.

When evaluating the sleep and psychology status by standard assessment scales, he scored 7 on the Pittsburg sleep quality index, 16 on the Epworth sleepiness scale, 52 on the self-rating anxiety scale, and 48 on the self-rating depression scale. An overnight polysomnography (PSG) test was performed immediately after his admission. The PSG data indicated a good night sleep, which had a total duration of 519.1 minutes, sleep efficiency of 86.2%, sleep latency of 50.5 minutes, and the ratio of REM sleep that reached 35.8%. The PSG data also indicated a moderate sleep breath disorder, of which the apnea-hypopnea index was 5.5, the average oxygen saturation (SaO_2_) was 94%, and the minimum SaO_2_ was 88%. The day after the PSG night, multiple sleep latency tests (MSLT) were performed. The MSLT data showed two periods of sleep-onset rapid eyes movement period across 4 successive tests; the average sleep latency was under 8 minutes, and the REM latency was under 7 minutes.

During hospitalization, a lumber puncture was performed, and the pressure of cerebrospinal fluid (CSF) was 120 mm H_2_O. The routine and cytology assessments of CSF were normal. The pathogen culture of CSF was negative. The protein of CSF increased to 834.5 mg/L, while the glucose and chlorides were normal. The level of CSF orexin and the allele of HLA-DRB1 gene were not detected.

**Figure 1:**
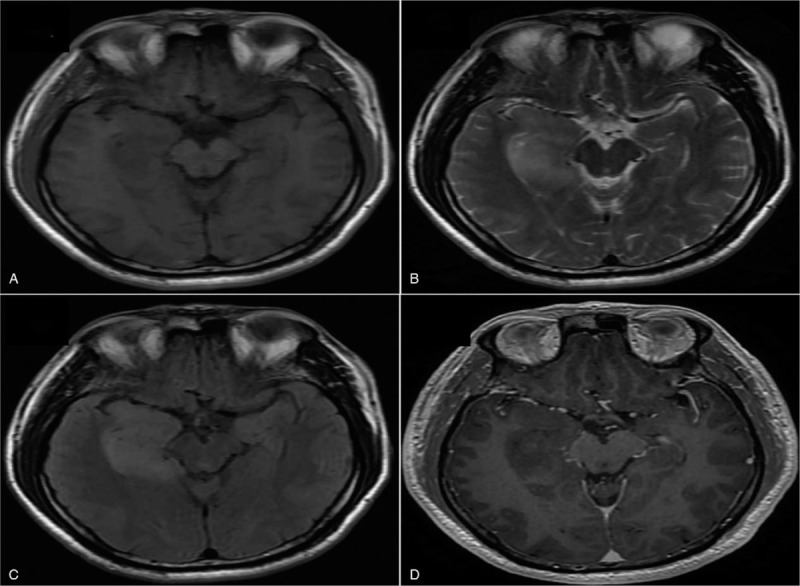
Magnetic resonance images of the case. A diffuse lesion located in hippocampal formation of right anteromedial temporal lobe without mass formed, with slight surrounding swelling, and disappearance of the sulci. The lesion displayed (A) hypointensity on T1-weighted images, hyperintensity on (B) T2-weighted images and (C) T2 fluid attenuated inversion recovery, and (D) nonenhancement.

**Figure 2:**
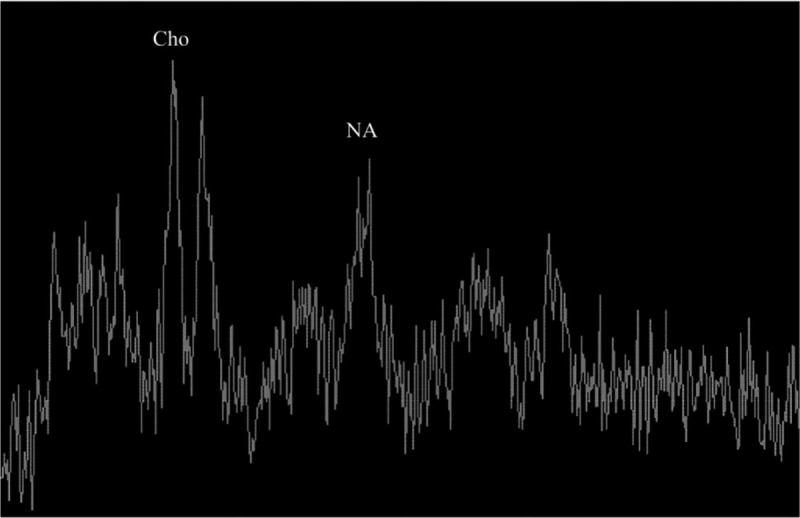
The magnetic resonance spectroscopy image of the case. It shows that the N-acetylaspartic acid level decreased, and the choline level increased.

**Figure 3:**
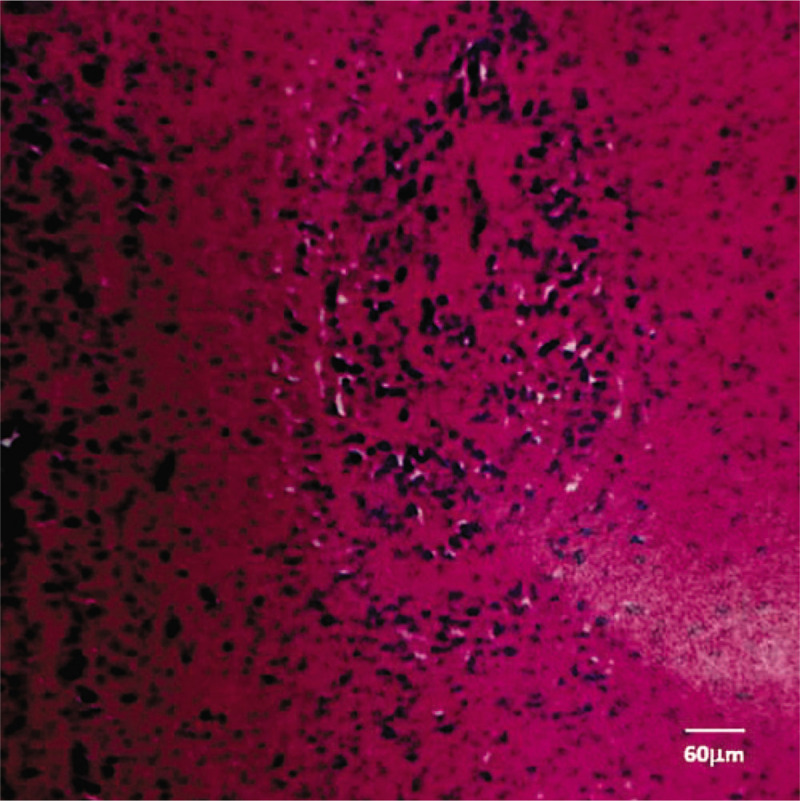
Histological section of the tumor. There were medium-sized, ovoid, long-protuberance, low-density tumor cells embedded in slim pink-stained collagen fibers and an amorphous eosinophilic matrix. Hematoxylin-eosin combined with immunohistochemical stain showed that the tumor was grade II glioma with anaplastic changes. Scale bar, 60 μm.

Magnetic resonance imaging showed an enlarged hippocampus area in the right anteromedial temporal lobe. The lesion showed hypointensity on T1-weighted images and hyperintensity on T2-weighted images and T2 fluid attenuated inversion recovery, which had nonenhancement (Fig. 1Fig. 1). Magnetic resonance spectroscopy of the lesion showed decreased N-acetylaspartic acid (NAA) levels and increased choline levels (Fig. 2). The lesion was surgically removed, and pathological examination was carried out. Histological section revealed medium-sized, ovoid-liked, long-protuberance, low-density tumor cells embedded in slim pink-stained collagen fibers and an amorphous eosinophilic matrix. Hematoxylin-eosin combined with immunohistochemical staining showed that the tumor was grade II glioma with anaplastic changes (Fig. 3).

According to the 3rd edition of the International Classification of Sleep Disorders (ICSD-3), we diagnosed the case as narcolepsy type 2. The ethics committee of the First People's Hospital of Chenzhou approved the study.

After surgical removal of the glioma from the hippocampus area of the right anteromedial temporal lobe, the patient's EDS symptoms disappeared immediately. He scored 3 on the Epworth sleepiness scale. During our follow-up 3 months later, he remained well with no complications.

## 3 Discussion

The patient showed clinical features of EDS lasting over 3 months. MSLT verified that the average sleep latency was less than 8 minutes and that there were at least 2 sleep-onset rapid eyes movement periods. There were no other EDS-causing reasons, such as sleep insufficiency, sleep breath disorder, restless leg syndrome, delayed sleep-wake phase disorder, drugs, or similar factors.^[7]^ According to the 3rd edition of the International Classification of Sleep Disorders (ICSD-3), we diagnosed the case with narcolepsy type 2.

We took particular interest in the relationship between the narcolepsy type 2 and its relevant glioma in the hippocampus area in this case. Although there have been no reports indicating that the glioma in the hippocampus area could cause narcolepsy, the association between the hippocampal formation and sleep has been mentioned in some studies. It has been reported that the volume of the hippocampus is related to the alertness and somnolence of patients with low-ventilation sleep apnea-hypopnea syndrome,^[4,5]^ and the broadening of the hippocampus fissure was found to be related to the severity of the sleep apnea-hypopnea syndrome.^[6]^ Studies of narcolepsy patients have shown the volume of the hippocampus to be significantly reduced in patients with cataplexy relative to those without cataplexy,^[8]^ and the atrophy of CA1 region in the hippocampus was found to be related to prolonged duration of somnolence and shortened latency of REM sleep.^[9]^ One study of Parkinson disease also showed that the volume of bilateral hippocampus and parahippocampus was larger in patient with EDS than in those without.^[10]^ PET of narcolepsy type 1 showed the hippocampus to be in a high metabolic state, which was positively related to the symptom of EDS.^[11]^ These studies may be consistent with our hypothesis that the lesion of the hippocampus was likely to be the cause of EDS.

The orexin projection system includes the lateral hypothalamus, reticular activating structure, and ventrolateral preoptic nucleus that were related to the pathogenesis of narcolepsy.^[3]^ Narcolepsy type 2 was diagnosed, and the EDS of the patient was relieved immediately after surgical removal of the glioma from the hippocampus area of the right anteromedial temporal lobe. In this way, glioma was considered the cause in this case. Based on our report of the relationship between the hippocampal glioma and narcolepsy type 2, the hippocampus might be another part of the regulation of the sleep-arousal pathway, and the glioma secretions might interact with the orexin projection system. Further studies of the pathogenesis should be performed.

## Author contributions

**Conceptualization:** Yuangao Liao.

**Data curation:** Yan He, You Yang, Xiaojie Li.

**Formal analysis:** Yan He, You Yang, Xiaojie Li.

**Funding acquisition:** Yuangao Liao, Fengzhen Huang.

**Investigation:** Yuangao Liao, Fengzhen Huang.

**Methodology:** Fengzhen Huang.

**Resources:** Yuangao Liao, Fengzhen Huang.

**Writing – original draft:** Yuangao Liao.

**Writing – review & editing:** Fengzhen Huang.
